# Effect of the Mahatma Gandhi National Rural Employment Guarantee Act (MGNREGA) on Malnutrition of Infants in Rajasthan, India: A Mixed Methods Study

**DOI:** 10.1371/journal.pone.0075089

**Published:** 2013-09-25

**Authors:** Manisha Nair, Proochista Ariana, Eric O. Ohuma, Ron Gray, Bianca De Stavola, Premila Webster

**Affiliations:** 1 National Perinatal Epidemiology Unit (NPEU), Nuffield Department of Population Health, University of Oxford, Oxford, United Kingdom; 2 Nuffield Department of Population Health, & Department of International Development, University of Oxford, Oxford, United Kingdom; 3 Nuffield Department of Obstetrics and Gynaecology, University of Oxford, Oxford, United Kingdom; 4 Department of Medical Statistics & Faculty of Epidemiology and Public Health, London School of Hygiene and Tropical Medicine, London, United Kingdom; 5 Nuffield Department of Population Health, University of Oxford, Oxford, United Kingdom; Aga Khan University, Pakistan

## Abstract

**Objectives:**

Analyse the effect of the Mahatma Gandhi National Rural Employment Guarantee Act (MGNREGA), a wage-for-employment policy of the Indian Government, on infant malnutrition and delineate the pathways through which MGNREGA affects infant malnutrition. Hypothesis: MGNREGA could reduce infant malnutrition through positive effects on household food security and infant feeding.

**Method:**

Mixed methods using cross-sectional study and focus group discussions conducted in Dungarpur district, Rajasthan, India. **Participants:** Infants aged 1 to <12 months and their mothers/caregivers. Final sample 528 households with 1056 participants, response rate 89.6%. Selected households were divided into MGNREGA-households and non-MGNREGA-households based on participation in MGNREGA between August-2010 and September-2011. **Outcomes:** Infant malnutrition measured using anthropometric indicators - underweight, stunting, and wasting (WHO criteria).

**Results:**

We included 528 households with 1,056 participants. Out of 528, 281 households took part in MGNREGA between August’10, and September’11. Prevalence of wasting was 39%, stunting 24%, and underweight 50%. Households participating in MGNREGA were less likely to have wasted infants (OR 0·57, 95% CI 0·37–0·89, p = 0·014) and less likely to have underweight infants (OR 0·48, 95% CI 0·30–0·76, p = 0·002) than non-participating households. Stunting did not differ significantly between groups. We did 11 focus group discussions with 62 mothers. Although MGNREGA reduced starvation, it did not provide the desired benefits because of lower than standard wages and delayed payments. Results from path analysis did not support existence of an effect through household food security and infant feeding, but suggested a pathway of effect through low birth-weight.

**Conclusion:**

Participation in MGNREGA was associated with reduced infant malnutrition possibly mediated indirectly via improved birth-weight rather than by improved infant feeding. Addressing factors such as lack of mothers’ knowledge and inappropriate feeding practices, over and above the social and economic policies, is key in efforts to reduce infant malnutrition.

## Introduction

In 2005–06, more than 40% of children in India were malnourished [1]. Malnutrition increases the risk of morbidity [2] and mortality among infants [3]. Macroeconomic and social policies can influence household income and poverty status which in turn function as social determinants of infant malnutrition [4]–[6]. The Mahatma Gandhi National Rural Employment Guarantee Act (MGNREGA) of Government of India targets deprivation and food insecurity in rural households [7], [8]. It is suggested that MGNREGA may have a general positive effect on the nutrition and well-being of children [9]. A recent study demonstrated that MGNREGA was associated with improved height-for-age among children 5–6 years of age [8]. However, the particular effect of MGNREGA on infant malnutrition is not known. We hypothesised that MGNREGA could reduce infant malnutrition through its positive effects on household food security and infant feeding. The aim of this study was to analyse the effect of MGNREGA on infant malnutrition and to delineate the pathways through which MGNREGA affects infant malnutrition. We found a positive effect of MGNREGA on infant nutrition mainly mediated through birth-weight.

## Methods

### Ethics Statement

Ethics approvals for the study were obtained from the Directorate of Medical, Health and Family Welfare Services, Government of Rajasthan, Jaipur and the University of Oxford Tropical Research Ethics Committee (OXTREC); OXTREC Reference: 43-11. Participant information sheet and consent form in local languages were used to elicit written informed consent from all participants before implementing the survey questionnaire. Written informed consent was taken from the mothers of infants before measuring the weight and length of infants. Written informed consent was also taken from all participants before the focus group discussions.

### Study Design

**Figure 1:**
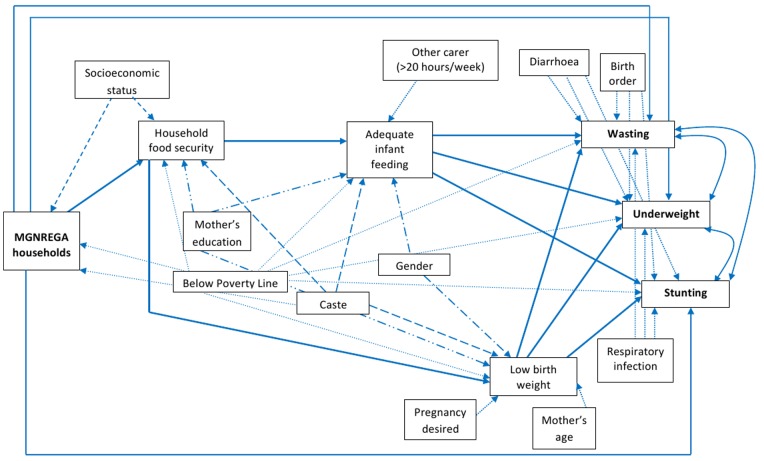
Hypothesized pathway of effect of MGNREGA on infant malnutrition.

MGNREGA is a wage-for-employment policy that “provides 100 days of guaranteed wage-employment to rural households whose adult members volunteer to do unskilled manual work” [10]. We conducted a literature review which suggested that MGNREGA as a source of income in the participating households could lead to food security, which would have a positive effect on infant feeding and thus, reduce their risk of malnutrition [10]–[12]. However, factors such as infant feeding practices and mothers’ knowledge about infant care and feeding could influence this pathway of effect [13]–[15]. Based on existing literature, a conceptual framework was developed to link MGNREGA and infant malnutrition (fig. 1), which guided the study design, data collection and analyses. We employed a mixed design i.e. a quantitative cross-sectional study and qualitative focus group discussions to analyse the effect and the mechanisms of effect of MGNREGA on infant malnutrition.

### Cross-sectional Study

MGNREGA is currently implemented in all districts in India, thus using an intervention trial to analyse its effect on infant malnutrition was not possible. The study was conducted in the Dungarpur district in the Indian state of Rajasthan where MGNREGA has been implemented since September, 2006. The degree of poverty and unemployment in the district alongside a high prevalence of malnutrition among children [16] makes it an appropriate setting for examining the potential benefits of MGNREGA.

The target sample size of 540 households for the cross-sectional study was calculated assuming a two-sided, two-sample comparison of proportions. The assumed baseline prevalence of being malnourished was based on the reported prevalence of the anthropometric indicators - underweight 41.7%, wasting 19.2% and stunting 53.4% in children under 5 years of age in Dungarpur district [17]. The minimum expected effect size was 13%, an effect demonstrated in the study by Uppal in Andhra Pradesh, India that measured the increase in the height-for-age of children (5–6 years) whose parents were employed through MGNREGA compared with their baseline (measurement before the implementation of MGNREGA) [8]. Equal sample size in each group (employed through MGNREGA or not) was assumed for the three anthropometric indicators at 5% significance level and 80% power. The sample size was adjusted by a variance inflation factor to account for the cluster sampling design [18] and further inflated by 5% to account for missing data.

Dungapur district is divided into five administrative blocks with 872 revenue villages in total. Each village was regarded as a cluster and 44 villages (which are 5% of total) were randomly selected using a single-stage cluster random sampling design. All households with infants aged 1 to <12 months (identified from the records of the local village nurse) in each of these villages were invited to participate. Infants <1 month of age were not included in the study because access to them was difficult due to cultural beliefs that restrict outsiders from coming in contact with infants until 40–45 days after birth. In total 615 households were approached and the response rate was 89.6%. The selected households were divided into MGNREGA-households and non-MGNREGA-households based on participation in the MGNREGA between August-2010 and September-2011.

A total of 551 households were surveyed with data collected on 1102 participants (551 pairs of mother and infant in each household). The survey questionnaire was administered face-to-face by MN and trained nurses to 551 mothers, and weight and recumbent length of 551 infants were measured. After excluding households with missing anthropometric data, 528 households with 1056 participants were included in the final analysis.

The three traditional anthropometric indicators of malnutrition – wasting, stunting and underweight were used as outcome variables. Wasting (low weight-for-height/length) is an indicator of “current nutritional status” and is an established measure of acute malnutrition [19]–[21]. Stunting is a measure of linear growth (low height/length-for-age) and reflects prolonged growth faltering [21], and underweight (low weight-for-age) is a composite indicator of wasting and stunting [21]. The recumbent length and weight of infants were measured using a portable infantometer (Seca 417, seca deutschland, Hamburg, Deutschland) and an electronic digital weighing scale (Seca 384, seca deutschland, Hamburg, Deutschland), respectively. The measurement methods were based on standard guidelines provided in the manual of the World Health Organisation’s (WHO) Multicentre Growth Reference Study [22]. The z-scores of weight-for-age, length-for-age and weight-for-length were calculated using the WHO 2007 STATA macro package (Statacorp, Texas, USA). Using a cut-off of less than minus two standard deviation (<−2SD) compared with the standard WHO-Multicentre Growth Reference Study population [23], the infants were categorised as underweight, stunted or wasted.

Household food security was measured using the standard Food and Nutrition Technical Assistance–2 (FANTA-2) household food insecurity indicator - Household Dietary Diversity Score [24]. ‘Adequate infant feeding practices’ was a composite variable of two indicators for assessing infant and young child feeding practices [25]. Definitions and descriptions of the 36 independent variables identified from literature as determinants of infant malnutrition and included in this study are provided in table 1.

**Table 1:** Definition and construct of the independent variables.

Sl. No	Variable name	Definition and construct
**Proximal determinants/biological factors**		
1	Diarrhoea	Defined as “three or more loose stools or any number of loose stools with blood in a twenty-four hour period” [35].
2	Upper respiratory tract infection (URTI)	Defined on the basis of elicited history from mothers on the presence of the following symptoms – cough and fever with or without rapid breathing.
**Intermediate determinants/behavioural factors**		
3	Adequate vaccination (age specific)	Vaccination history of the infants was matched with their age and based on the Universal Immunisation Programme schedule used in India, the infants were classified as having received adequate or inadequate (age specific) vaccination.
4	Early initiation of breast feeding	“Proportion of children born in the last 24 months who were put to the breast within one hour of birth” (WHO’s indicators for assessing infant and young child feeding practices [25]).
5	Adequate infant feeding practices	Composite variable of two indicators for assessing infant and young child feeding practices [25]:Infants’ under 6 months - Exclusive breastfeeding defined as “Proportion of infants 0–5 months of age who are fed exclusively with breast milk.” Infants’ ≥6 months - Minimum acceptable diet defined as “Proportion of children 6–23 months of age who receive a minimum acceptable diet (apart from breast milk).” The variable includes information on minimum meal frequency and minimum dietary diversity.
6	Cleanliness of water and food *(Boiled water* *given to infants and Proper disposal* *of infant faeces)*	Mothers were asked about the type of water given to infants and about the methods used to dispose infant stool. Based on the criteria for adequate method of faeces disposal provided by the WHO’s Core questions on drinking-water and sanitation for household surveys [36], the data was divided into adequate and inadequate.
7	Infant care *(Duration of care by other carers, Adequate feeding during diarrhoea and Oral Rehydration Salt to infants with diarrhoea)*	The continuous variable - total hours infant was cared for by someone other than the mother in the past week was categorised into “none”, ≤20 hours per week and >20 hours per week. The cut-off was based on the mean duration of care provided by the other carers = 19.5 (±0.63) hours per week in the sampled households. Infant feeding during episodes of diarrhoea was classified as adequate and inadequate based on a score generated using the District Level Household Survey tool [37]. Mothers were also asked if Oral Rehydration Salt was given to the infants during diarrhoea.
8	Health seeking behaviour *(Treatment for diarrhoea/URTI, Mother had antenatal* *checkups, Institutional delivery and* *Household at least one sibling died)*	The infants were considered treated for diarrhoea/URTI if they were taken to a doctor/public or private hospital/village nurse. Mothers were asked whether they received any antenatal checkup during their pregnancy with the index child (child participating in the study). Place of delivery was enquired and classified as institutional or home delivery. The mothers were asked about the total number of children they have and if any child died.
**Intermediate determinants/socio-cultural factors**		
9	Caste	Based on the social class system in India the households were divided into two groups – schedule caste and/or schedule tribe and other social class. Scheduled castes comprise of the social groups that has suffered the greatest burden of deprivation within the caste system and were regarded as untouchables [38]. Scheduled tribes include approximately 700 officially recognized social groups that have historically been geographically and socially isolated [39]. Other castes were those that did not belong to either of these groups and were presumably better off in their social status.
**Distal determinants/structural factors**		
10	Access to safe drinking water and Proper sanitation	Sources of drinking water and sanitation/toilet facilities were categorised as adequate/proper and inadequate/improper based on the WHO’s Core questions on drinking-water and sanitation for household surveys [36].
11	Crowding/household density	Defined as total number of members (physically living in the household) per room.
12	Socio-economic status	The Demographic and Health Survey [40] instrument was used to elicit information about the household amenities and assets including landownership and domestic animals which were then weighted using the factor loadings from a principal component analysis of the asset variables. The calculated scores of each asset variable were added to generate an asset index – a continuous variable which was then divided into quintiles. The lowest two quintiles were combined to construct four categories of the socioeconomic status variable. The method used is as suggested by the World Bank for calculating asset and wealth indices [41].
13	Mothers’ level of education, Fathers’level of education	Based on the reported history from the mother/caregiver, the education levels of mothers and fathers of infants were divided into – illiterate corresponding to no formal education, primary education if they completed primary school and secondary or higher education. Some mothers were taught to sign their names, but they did not know how to read or write, such mothers were categorised as illiterate.
14	Mother worked after delivery	Information was elicited with regards to whether the mothers of the index child worked to earn money after delivery and was classified as “not worked”, “worked in MGNREGA” and “employed in other jobs”. If the mothers worked in the family’s farm without pay, they were not considered as employed.
15	Primary occupation of household	Primary occupation of the household was elicited by enquiring about the profession of each adult member of the household. If any member had a regular job, the household was included under “regular occupation” and if all members were engaged in seasonal employment (agriculture/agriculture labours), the household was included under “seasonal occupation”.
16	Received food through the PublicDistribution System	Whether the household received food from the Public Distribution System which facilitates the supply of food grains to the poor households at a subsidised price in India [42].
17	Below poverty line households	This was based on the availability of the below poverty line status card for the household.The Planning Commission of India defines “Below Poverty Line” households as households (average 5 family members) with per capita consumption expenditure of INR 672.8 on a monthly basis in rural areas and INR 859.6 in urban areas at prices prevailing in 2009–10 [30].
18	Household food security	Measured as household dietary diversity score generated using the Food and Nutrition Technical Assistance–2 (FANTA-2) household food insecurity indicator - Household Dietary Diversity Score (HDDS) [24].
**Empowerment of mothers of infants**		
19	Mothers participating in household decision making, Decision on spending mother’s earning, Decision on spending husband’s earning	The Demographic and Health Survey tool [40] for measuring women empowerment was used and three categorical variables each assessing the role of women in different types of household decision making were created.
**Demographic factors**		
20	Child’s age, Mother’s age	Child’s and mother’s age was noted from the records of the village nurse and the community and nutrition health workers.
21	Gender of the child	The gender of the infant was noted as male or female.
22	Low birth weight	Reported birth weight (verified using records from village nurses and community health and nutrition workers where available) was categorised as <2500 grams and ≥2500 grams according to the definition of Low birth-weight given by WHO, which is “weight at birth of less than 2500 grams (5.5 pounds)”.
23	Pregnancy desired	Mothers were asked whether they desired to have the index child.
24	Presently pregnant	Mothers were asked whether they were pregnant at the time of the survey.
25	Birth order of the infant	Birth order was calculated based on the number of children born alive prior to the index child and was categorised as first born, second born, third born and 4+ live births. This did not include still births and abortions.
26	Religion	All participants were asked about the religion that the members of the household primarily followed.

### Focus Group Discussions

Focus groups were used to generate themes and interactions through group discussion with the mothers of infants to explore the proposed mechanisms of effect of MGNREGA on infant malnutrition. A purposive sampling method based on mothers’ willingness to participate was used to recruit participants for eleven focus group discussions (two in each of the five administrative blocks and one pilot study). Participants comprised of 62 mothers of infants who were a sub-set of the randomly selected participants for the cross-sectional study (a nested sampling approach). The focus group discussions were conducted by MN with the help of a trained nurse using a semi-structured topic guide. The discussions were recorded, transcribed non-verbatim and translated into English. The transcripts were managed using the QSR International’s NVivo9 software and analysed using set and emergent themes.

### Statistical Analyses

Descriptive analyses of all variables were conducted. Univariable logistic regression analyses were done for each of the three binary anthropometric outcomes – underweight, wasting and stunting to estimate the crude odds ratios. Since participation in MGNREGA was not randomised, exploratory logistic regression analysis was also used to identify the factors that influenced households’ participation in MGNREGA. These factors along with other known confounders were then entered into the multivariable logistic regression models to estimate the adjusted effects of households’ participation in MGNREGA on the anthropometric indicators of malnutrition. Tests for interaction were conducted to identify the factors that could modify the effect of MGNREGA on the outcome variables. To account for data dependency and within-cluster correlations, robust standard errors (Huber-White sandwich estimator) were reported.

Path analysis was performed to quantify the hypothetical pathways (shown in fig. 1) by fitting a set of regression equations under the assumption that the model is not affected by unmeasured confounding [26]. Weighted least square adjusted for mean and variance was used to estimate the parameters of the model [27]. Three model fit indices, Chi square(χ^2^) test for model fit, Comparative Fit Index (CFI) and Root Mean Square Error of Approximation (RMSEA), each related to a specific aspect of the model were used to quantify the degree of correspondence between the hypothesized models and the data [28], [29]. Indirect effects were computed by multiplying the relevant path coefficients. Statistical significance was considered at the 5% level and the analyses were performed using STATA version 11 (Statacorp, Texas, USA) and Mplus version 7.

## Results

Of the total 528 households, 53% (95% Confidence Interval (CI) = 48.9–57.5; n = 281) participated in MGNREGA. The overall prevalence of underweight, stunting and wasting among the infants in the study households was 50.4% (95% CI = 46.0–54.7), 24.4% (95% CI = 20.8–28.3) and 39% (95% CI = 34.8–43.3), respectively. The characteristics of the study population are presented in table 2. More households employed through MGNREGA belonged to the lower socioeconomic status, were categorised as below poverty line households (as defined by the Planning Commission of India [30]) and were engaged in seasonal employment compared to non-MGNREGA households. Mean household density was higher in the MGNREGA-households and access to proper sanitation was lower compared to the non-MGNREGA households. Apart from these factors, the MGNREGA and non-MGNREGA households did not differ in other socio-demographic characteristics including household food security (table 3).

**Table 2:** Description of the study population.

Characteristics	Number ofhouseholds	*Proportion of households in % (95% Confidence Interval)
**Non-health policy: MGNREGA** ^ƒ^		
Employed through MGNREGA between Aug’10 and Sep’11	281	53.2 (48.9, 57.5)
**Outcome – Malnutrition**		
Infant underweight (<−2SD of WAZ)	266	50.4 (46, 54.7)
Infant wasted (<−2SD of WLZ)	206	39 (34.8, 43.3)
Infant stunted (<−2SD of LAZ)	129	24.4 (20.8, 28.3)
**Proximal determinants/Biological factors**		
Infant having diarrhoea	79	15 (12, 18.3)
Infant having Upper Respiratory Tract Infection (URTI)	49	9.3 (6.9, 12.1)
**Intermediate determinants/Behavioural factors**		
Infant with adequate vaccination (age specific vaccination)	207	39.2 (35, 43.5)
Adequate infant feeding	122	23.1 (19.6, 26.9)
Cleanliness of water and food		
Boiled water given to infants [Households infants given water, n = 380]	7	1.8 (0.7, 3.8)
Baby utensils washed with hot water [Households utensils used, n = 393]		
No	254	64.6 (59.7, 69.4)
Yes	139	35.4 (30.6, 40.3)
Proper disposal of infant faeces	23	4.4 (2.8, 6.5)
Health seeking for infants		
Treatment for diarrhoea/respiratory infections [n = 119]	84	70.6 (61.5, 78.6)
At least one sibling died	38	7.2 (5.1, 9.7)
Institutional delivery	427	80.9 (77.3, 84.1)
Infant care		
Duration of care per week by other carers		
None	242	45.8 (41.5, 50.2)
≤20 hours	169	32.0 (28.0, 36.2)
>20 hours	117	22.2 (18.7, 25.9)
Adequate feeding of infants during diarrhoea (households in which children had diarrhoea; n = 79)	4	5.1 (1.4, 12.5)
ORS^†^ given ((households in which childrenhad diarrhoea; n = 79)	29	36.7 (26.1, 48.3)
**Intermediate determinants/Socio-cultural factors**		
Caste		
Non Schedule Caste/Schedule Tribe	120	22.7 (19.2, 26.5)
Schedule Caste/Schedule Tribe	408	77.3 (73.5, 80.8)
Childs’ gender		
Male	283	53.6 (49.2, 57.9)
Female	245	46.4 (42.1, 50.8)
**Distal determinants/structural factors**		
Safe source of drinking water	459	86.9 (83.8, 89.7)
Proper sanitation	29	5.5 (3.7, 7.8)
Primary occupation		
Seasonal	424	80.3 (76.7, 83.6)
Regular	104	19.7 (16.4, 23.3)
Below poverty line (BPL)	415	78.6 (74.9, 82.0)
Received food from Public distribution system (PDS)	424	80.3 (76.7, 83.6)
Socio-economic status (Asset index)		
Lowest 2 quintiles	212	40.2 (35.9, 44.5)
Third quintile	105	19.9 (16.6, 23.6)
Fourth quintile	106	20.1 (16.7, 23.8)
Fifth quintile	105	19.9 (16.6, 23.6)
Mothers’ level of education		
Illiterate	349	66.1 (61.9, 70.1)
Primary education	141	26.7 (22.9, 30.7)
Secondary and higher	38	7.2 (5.1, 9.7)
Fathers’ level of education		
Illiterate	182	34.5 (30.4, 38.7)
Primary education	236	44.7 (40.4, 49.1)
Secondary and higher	110	20.8 (17.4, 24.6)
**Empowerment of mothers of infants**		
Mothers participating in household decision making	14	2.7 (1.5, 4.4)
Mothers who own household property	1	0.2 (0, 1.1)
Decision on spending mother’s earning (households where mother of infant earns; n = 76)		
Inlaws	36	47.4 (35.8, 59.2)
Mother of infant	4	5.3 (1.5, 12.9)
Husband	22	28.9 (19.1, 40.5)
Jointly by mother of infant and her husband	14	18.4 (10.5, 29.0)
Decision on spending husband’s earning (households where husband earns; n = 525)		
Inlaws	247	47 (42.7, 51.4)
Mother of infant	15	2.9 (1.6, 4.7)
Husband	178	33.9 (29.9, 38.1)
Jointly by mother of infant and her husband	85	16.2 (13.1, 19.6)
**Demographic characteristics**		
Child’s age		
1 to <6 months	230	43.6 (39.3, 47.9)
≥6 to <12 months	298	56.4 (52.1, 60.7)
Mothers’ age		
≤20 years	38	7.2 (5.1, 9.7)
21–25 years	266	50.4 (46.3, 54.7)
26–30 years	172	32.6 (28.6, 36.8)
>30 years	52	9.8 (7.4, 12.7)
Low birth weight infants [birth-weight<2500 grams; missing data = 121]	233	44.1 (39.8, 48.5)
Pregnancy was desired	509	96.4 (94.4, 97.8)
Mother is presently pregnant	20	3.8 (2.3, 5.8)
Birth order		
First	142	26.9 (23.2, 30.9)
Second	166	31.4 (27.5, 35.6)
Third	124	23.5 (19.9, 27.3)
≥four	96	18.2 (14.9, 21.7)
Language		
Wagri	504	95.5 (93.3, 97.1)
Hindi	9	1.7 (0.8, 3.2)
Gujarati	11	2.1 (1.0, 3.7)
Banjari	4	0.7 (0.2, 1.9)
Religion (Hindu)	528	100 (99.3, 1.0)**

* Total number of households = 528 (unless specified along with the variable); ƒMGNREGA - Mahatma Gandhi National Rural Employment Guarantee Act; †ORS - Oral rehydration salt;

** one-sided 97.5% confidence interval.

**Table 3:** Prevalence of malnutrition and the determinants of malnutrition in MGNREGA & non-MGNREGA households.

	Frequency of household (%)		
Determinants of malnutrition	Non-MGNREGA^ƒ^	MGNREGA^ƒ^	P-value (Chi square test)
**Outcome (Anthropometric indicators of malnutrition)**			
Wasting	108 (52.4)	98 (47.6)	0.038
Stunting	66 (51.2)	63 (48.8)	0.251
Underweight	139 (52.3)	127 (47.7)	0.011
**Proximal Determinants**			
Diarrhoea	42 (53.2)	37 (46.8)	0.217
Upper respiratory tract infection (URTI)	24 (49)	25 (51)	0.746
**Intermediate determinants/behavioural factors**			
Adequate vaccination (age specific)	101 (48.6)	107 (51.4)	0.509
Early initiation of breast feeding	193 (46.6)	221 (53.4)	0.887
Adequate infant feeding	56 (45.9)	66 (54.1)	0.824
*Cleanliness of water and food*			
Boiled water given to infants [Households infants given water, n = 381]	4 (57.1)	3 (42.9)	0.580
Baby utensils washed with hot water [households utensils used, n = 393]	72 (51.8)	67 (48.2)	0.143
Proper disposal of infant faeces	15 (65.2)	8 (34.8)	0.070
*Health seeking behaviour*			
Treatment for diarrhoea/URTI [n = 119]	43 (51.2)	41 (48.8)	0.981
Institutional delivery	196 (45.9)	231 (54.1)	0.405
Household at least one sibling died	16 (42.1)	22 (57.9)	0.549
*Infant care*			
Cared by someone other than the mother	124 (43.4)	162 (56.6)	0.087
Duration of care by other carers			
None	123 (50.8)	119 (49.2)	
≤20 hours per week	88 (52.1)	81 (47.9)	<0.001
>20 hours per week	36 (30.8)	81 (69.2)	
Adequate feeding during diarrhoea (children with diarrhoea; n = 79)	3 (75.0)	1 (25.0)	0.369
ORS† given (children who had diarrhoea; n = 79)	13 (44.8)	16 (55.2)	0.258
**Intermediate determinants/socio-cultural factors**			
Caste			
Non- Schedule Tribe/Schedule Caste	64 (53.3)	56 (46.7)	0.102
Schedule Tribe/Schedule Caste	183 (44.9)	225 (55.1)	
Gender of the child			
Male	133 (47.0)	150 (53)	0.915
Female	114 (46.5)	131 (53.5)	
**Distal determinants/structural factors**			
Access to safe source of drinking water	213 (46.4)	246 (53.6)	0.656
Proper sanitation	19 (65.5)	10 (34.5)	0.038
Crowding [mean household density (SE), t-statistic]	3.7 (0.11)	4.1 (0.12)	0.054*
Socio-economic status (Asset index)			
Lowest 2 quintiles	94 (44.3)	118 (55.7)	
Third quintile	44 (41.9)	61 (58.1)	
Fourth quintile	46 (43.4)	60 (56.6)	0.025
Fifth quintile	63 (60)	42 (40)	
Mothers’ level of education			
Illiterate	156 (44.7)	193 (55.3)	
Primary education	69 (48.9)	72 (51.1)	0.252
Secondary and higher	22 (57.9)	16 (42.1)	
Fathers’ level of education			
Illiterate	86 (47.2)	96 (52.8)	
Primary education	108 (45.9)	128 (54.1)	0.904
Secondary and higher	53 (48.2)	57 (51.8)	
Primary occupation			
Seasonal	190 (44.8)	234 (55.2)	0.067
Regular	57 (54.8)	47 (45.2)	
Received food through Public distribution system (PDS)	189 (44.6)	235 (55.4)	0.040
Below poverty line (BPL) households	176 (42.4)	239 (57.6)	<0.001
Household dietary diversity score [mean (SE), t-statistics]	5.8 (0.10)	5.7 (0.10)	0.251
**Empowerment of mothers of infants**			
Mothers participating in household decision making	8 (57.1)	6 (42.9)	0.431
Decision on spending mother’s earning (households where mother of infant earns; n = 74)			
Inlaws	6 (16.7)	30 (83.3)	
Mother of infant	2 (50.0)	2 (50.0)	0.292
Husband	3 (13.6)	19 (86.4)	
Jointly by mother of infant and her husband	4 (28.6)	10 (71.4)	
Decision on spending husband’s earning (households where husband earns; n = 525)			
Inlaws	120 (48.6)	127 (51.4)	
Mother of infant	8 (53.3)	7 (46.7)	0.339
Husband	74 (41.6)	104 (58.4)	
Jointly by mother of infant and her husband	44 (51.8)	41 (48.2)	
**Demographic variables**			
Live births [mean (SE), t-statistics]	2.4 (0.08)	2.4 (0.08)	0.884*
Low birth weight			
No	72 (41.4)	102 (58.6)	
Yes	113 (48.5)	120 (51.5)	0.194
Missing	62 (51.2)	59 (48.8)	
Mother’s age [mean (SE), t-statistics]	26.1 (0.27)	25.3 (0.23)	0.0271*
Child’s age [mean (SE), t-statistics]	6.8 (0.20)	6.4 (0.19)	0.195*

Total sample = 528 (unless specified along with the variable); ƒMGNREGA - Mahatma Gandhi National Rural Employment Guarantee Act;

† ORS - Oral rehydration salt;

* P-value for t-statistics.

Further, the results of the exploratory logistic regression modelling conducted to elucidate systematic differences leading to MGNREGA participation and non-participation that may be brought about by factors such as socioeconomic status, belonging to below poverty line status, parents’ level of education, caste, household food security, households’ enrolment in Public Distribution system, primary occupation of the household and indicators for health seeking behaviour (treatment for diarrhoea, institutional delivery, household at least one sibling died and adequate vaccination) suggested that only households categorised as being below the poverty line were more likely to participate in the programme (OR = 1.98; 95% CI = 1.22–3.19; p = 0.006). Apart from this no other known factor was found to be significantly associated with participation in MGNREGA.

### Effect on Malnutrition

The adjusted odds of infants being underweight and wasted in households participating in MGNREGA were respectively 52% (adjusted OR = 0.48; 95% CI = 0.30–0.76, p = 0.002) and 43% (OR = 0.57; 95% CI = 0.37–0.89; p = 0.014) lower compared with households that did not participate in MGNREGA after controlling for socioeconomic status, below poverty line, caste, duration of care by other carers, birth order and child’s age (table 4). There was no statistically significant difference in infant stunting between households participating in MGNREGA and those not (adjusted OR = 0.70; 95% CI = 0.46–1.05; p = 0.086).

**Table 4:** Effect of MGNREGA on underweight, wasting and stunting.

Determinants of malnutrition	Underweight			Wasting				Stunting		
	aOR (95% CI)	P-value (Wald test)	P-value (Trend)	aOR (95% CI)	P-value (Wald test)		P-value (Trend)	aOR (95% CI)	P-value (Wald test)	P-value (Trend)
**Employed through MGNREGA (Non-health Policy)**										
No	1	–		1	–			1	–	
Yes	0.48 (0.30–0.76)	0.002	NA	0.57 (0.37–0.89)	0.014		NA	0.70 (0.46–1.05)	0.086	NA
**Distal determinants/structural factors**										
Socio-economic status										
Lowest 2 quintiles	1	–		1				1	–	
Third quintile	0.72 (0.48–1.06)	0.096		0.62 (0.35–1.11)	0.108			0.86 (0.47–1.58)	0.627	
Fourth quintile	0.62 (0.34–1.11)	0.107	0.002	0.86 (0.52–1.45)	0.573		0.148	0.91 (0.52–1.58)	0.733	0.001
Fifth quintile	0.25 (0.11–0.54)	0.001		0.58 (0.31–1.11)	0.098			0.17 (0.08–0.39)	<0.001	
Below poverty line										
No				1	–					
Yes	Not significant for the model			1.65 (0.93–2.91)	0.083		NA	Not significant for the model		
**Intermediate determinants/socio-cultural factors**										
Caste										
Non-ST/SC^†^	1	–		1	–					
ST/SC^†^	2.72 (1.27–5.80)	0.011	NA	2.83 (1.39–5.76)	0.005		NA	Not significant for the model		
**Intermediate determinants/behavioural factors**										
Duration of care by other carers										
None	1	–		1	–			1	–	
≤20 hours per week	0.84 (0.55–1.28)	0.407	0.173	1.20 (0.75–1.93)	0.245		0.208	0.80 (0.51–1.25)	0.323	0.743
>20 hours per week	1.52 (0.94–2.46)	0.088		1.36 (0.83–2.22)	0.222			1.17 (0.74–1.86)	0.499	
**Demographic factors**										
Birth order of the infant										
First	1	–		1	–			1	–	
Second	1.14 (0.70–1.86)	0.602		1.20 (0.67–2.14)	0.533			1.93 (0.97–3.84)	0.062	
Third	1.55 (0.89–2.71)	0.116	0.004	1.60 (0.84–3.04)	0.084		0.019	1.51 (0.75–3.06)	0.241	0.058
≥Four	1.85 (1.14–2.99)	0.014		2.11 (1.15–3.86)	0.011			2.04 (0.97–4.28)	0.060	
Child’s age										
<6 months	1	–		1	–			1	–	
≥6 months	1.62 (1.05–2.51)	0.030	NA	1.64 (1.10–2.45)	0.017	NA		1.76 (1.04–2.99)	0.037	NA

Total sample = 528 households, aOR – adjusted odds ratio, CI – Confidence Interval, ST/SC^†^ - Schedule Caste/Schedule Tribe, NA – Not applicable.

### Mechanisms of Effect

Four themes emerged from the focus group discussions to help explain the quantitative findings relating MGNREGA to infant malnutrition. These included the effect of MGNREGA on household food security, impact of poverty on infant feeding, mothers’ knowledge of infant feeding and cultural factors affecting infant feeding.

#### MGNREGA’s effect on household food security

Agriculture is the main source of livelihood in the district of Dungarpur. The participants suggested that earnings from MGNREGA contributed towards preventing hunger and starvation when there was crop failure particularly among the poor.

*“If there is no Rojgar Guarantee [MGNREGA] then what do we eat? If we get some money [we] can buy food grains for the house and [we] can go on. What ripens in cultivation? What do we eat? There are no crops, so we have benefited from Rojgar [MGNREGA].” (M-13)*

A participant employed through MGNREGA at the time of interview commented that if the programme was to be abolished, the poor people would be the losers because they do not have recourse to any other means of income. Although it was agreed that MGNREGA conferred some benefits in terms of preventing hunger during crop failure and meeting minor household expenses, the participants complained of low wages and delays in receiving payments. They did not trust the supervisors responsible for paying the wages to the workers, and commented that they siphoned off money and paid only a small portion of the daily wages to the workers. The participants perceived these as barriers to receiving the complete financial benefits from the programme.

*“Who gets 100 [INR]? The person responsible for paying us the wages takes away most of it. Last time I got 200 rupees for 11 days.” (M-19)*

#### Influence of poverty on infant feeding

Poverty itself was a factor that negatively affected infant feeding. “Inability to afford” compromised the type of complementary foods given to the infants. These were mostly biscuits or a piece of dry *roti* (bread) made of either wheat or maize. Although the mothers were aware that these foods could harm the child, they could not afford to buy anything else. For example, a mother mentioned that her young daughter was unable to swallow dry bread and often vomited it out, so she had no option other than breastfeeding.

#### Mothers’ knowledge of infant feeding

Mothers’ knowledge of initiation of breastfeeding, exclusive breastfeeding and weaning appeared to be inadequate. There were several misconceptions such as breast milk is produced only after two hours of delivery of the child, and inappropriate practices such as giving water and animal milk (cow’s, goat’s, buffalo’s) with or without *ghee* (clarified butter) to infants <6 months.

*“I started breastfeeding after 2 hours because I did not have enough milk. Milk comes only after 2 hours.” (M-40)*

#### Cultural factors affecting infant feeding

In addition to poverty, cultural practices appeared to inform mothers’ knowledge and influenced the infant feeding practices. Apart from initiation of breastfeeding and exclusive breastfeeding, the time of initiation of complementary feeding and the type of food given was also determined by social customs.

*“We have this custom, Mama [maternal uncle] will give food to the child, rice, in 8th month or 9th month or 11th month. There is a puja [worship God] and then rice is given to the child.” (M-51)*

### Pathways of Effect

**Figure 2:**
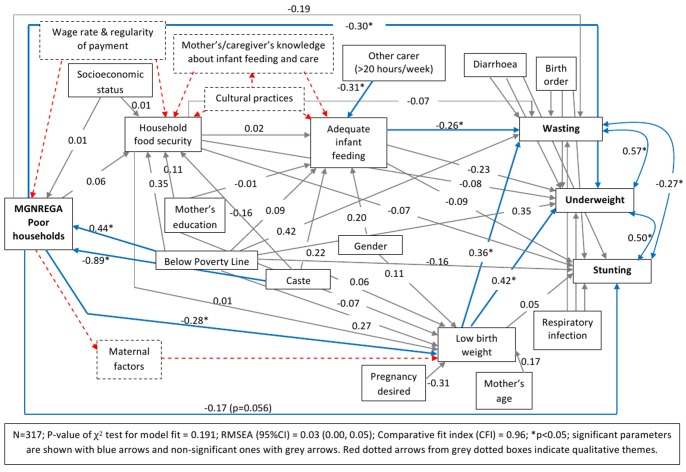
Path model showing the effect of MGNREGA on infant malnutrition in POOR households.

**Figure 3:**
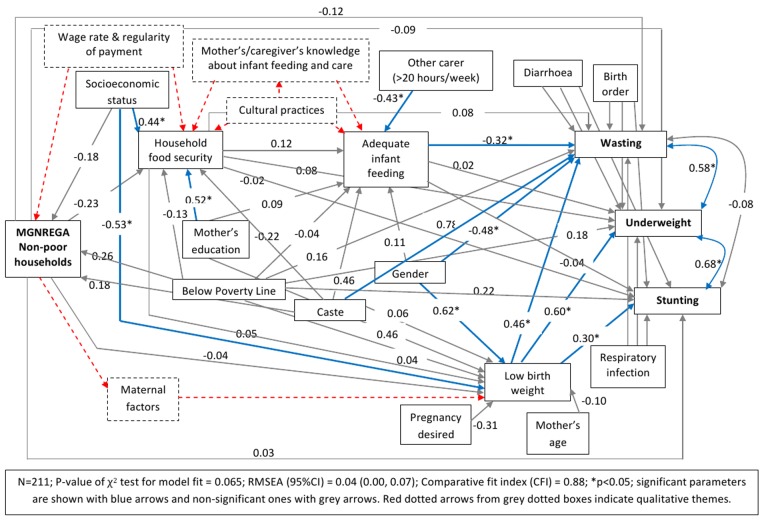
Path model showing the effect of MGNREGA on infant malnutrition in NON-POOR households.

We hypothesized *a priori* that MGNREGA will affect infant malnutrition through improving household food security and via this improve infant feeding and birth weight (fig. 1). Using path analysis we were able to estimate the effects of MGNREGA on infant malnutrition (wasting, underweight and stunting) controlling for potential confounders. Models’ assessment revealed that there was a direct pathway from MGNREGA to birth weight (i.e. not just via household food security) that had not been initially hypothesised. Further, because we found evidence that “socioeconomic status” might modify the effect of MGNREGA on household food security, we fitted separate path models to subgroups of the participants defined by their wealth index (those in the lowest 3 quintiles, the poor households, n = 317, separately from those in the top two quintiles, the non-poor households n = 211). Five models (three models with single outcome variable – wasting or stunting or underweight, and two models with all three outcomes with and without households with missing data) for each of the two sample groups (poor and non-poor) were fitted and compared using the fit indices. Robust estimates of standard error were used to take cluster sampling into account. The models with the best fit are presented in figures 2 and 3 (the model fit indices are given in the figures).

In the poor-MGNREGA model, the estimated effects of MGNREGA via household food security and adequate infant feeding on wasting, underweight and stunting were each 0.00 (p-value = 0.73) (table 5). However, there was evidence of a significant pathway of effect of MGNREGA on wasting and underweight through “low birth-weight” (estimated effect for wasting was −0.10 (Standard Error (SE) = 0.05); p = 0.047 and underweight was −0.12 (SE = 0.05); p = 0.033). No such effect was observed for stunting (table 5). In addition to the effect via low birth-weight, MGNREGA was found to have a direct negative effect on underweight, but not on wasting (table 5) suggesting that there could be other unidentified variables (not included in our models) in the pathway between MGNREGA and underweight. Although there was no effect on stunting via the low birth weight pathway (table 5), a direct negative effect was estimated (−0.17; SE = 0.09; p = 0.056), also suggesting a possible role of other unidentified factors (fig. 2).

**Table 5:** Estimates of MGNREGA’s pathways of effect on wasting, underweight and stunting in poor and non-poor households.

Poor Households						
Effects	Effects from MGNREGA to Wasting		Effects from MGNREGA to Underweight		Effects from MGNREGA to Stunting	
	Estimate (SE)	P-value	Estimate (SE)	P-value	Estimate (SE)	P-value
**Total-total**	−0.31 (0.12)	0.008	−0.43 (0.16)	0.007	−0.19 (0.09)	0.043
**Total direct**	−0.19 (0.11)	0.079	−0.30 (0.14)	0.032	−0.17 (0.09)	0.056
**Total indirect**	−0.11 (0.06)	0.060	−0.13 (0.06)	0.036	−0.02 (0.03)	0.467
**Specific indirect**						
Via adequate feeding	−0.01 (0.03)	0.806	−0.01 (0.03)	0.805	−0.00 (0.01)	0.821
Via low birth weight	−0.10 (0.05)	0.047	−0.12 (0.05)	0.033	−0.01 (0.02)	0.597
Via household food security	−0.00 (0.01)	0.577	−0.01 (0.01)	0.560	−0.00 (0.01)	0.609
Via household food security and adequate feeding	0.00 (0.00)	0.735	0.00 (0.00)	0.738	0.00 (0.00)	0.744
Via household food security and low birth weight	0.00 (0.00)	0.905	0.00 (0.00)	0.903	0.00 (0.00)	0.905
**Non-poor Households**						
**Total-total**	−0.13 (0.14)	0.304	−0.14 (0.17)	0.404	0.02 (0.14)	0.904
**Total direct**	−0.12 (0.13)	0.368	−0.09 (0.16)	0.550	0.03 (0.14)	0.853
**Total indirect**	−0.02 (0.07)	0.730	−0.05 (0.08)	0.548	−0.01 (0.04)	0.822
**Specific indirect**						
Via adequate feeding	0.01 (0.04)	0.756	−0.00 (0.01)	0.906	0.00 (0.01)	0.783
Via low birth weight	−0.02 (0.06)	0.744	−0.03 (0.08)	0.744	−0.01 (0.04)	0.752
Via household food security	−0.02 (0.02)	0.388	−0.02 (0.02)	0.341	0.01 (0.02)	0.816
Via household food security and adequate feeding	0.01 (0.01)	0.213	−0.00 (0.00)	0.892	0.00 (0.00)	0.738
Via household food security and low birth weight	−0.01 (0.01)	0.543	−0.01 (0.01)	0.543	−0.00 (0.01)	0.523

SE – Standard error.

Unlike the poor–MGNREGA model, neither the direct nor the indirect estimated effects of MGNREGA were found to be statistically significant in the non-poor-MGNREGA model (fig. 3). However, after adjusting for other variables, a similar effect on wasting and underweight was observed through negative effects on low birth-weight, but the variable at the distal end was socioeconomic status instead of MGNREGA (fig. 3). The estimated specific indirect effect of higher socioeconomic status via low birth-weight on wasting was −0.27 (SE = 0.12); p = 0.023 and on underweight was −0.35 (SE = 0.16); p = 0.028. The estimated coefficients for the effects of MGNREGA on wasting, underweight and stunting for the poor and non-poor models are provided in table 5.

As shown in table 2, information on birth-weight of infants was missing in 121 households due to non-availability of birth records for these infants from the health centres. However, we found the households with missing observations to be evenly distributed between the MGNREGA and the non-MGNREGA groups (MGNREGA group = 59 households (21% of the total MGNREGA households) and non-MGNREGA group = 62 households (25% of the total non-MGNREGA households); p-value for χ2 test = 0.263). The reason for this missing data was non-availability of recorded birth-weights which cannot be attributed to any specific characteristics of the sample population. Further, the estimator weighted least square controlled for mean and variance (used in this study) with pair-wise deletion is considered to be an efficient and unbiased estimator for models with missing data [31]. Nevertheless, bias due to missing data cannot be completely ruled out.

## Discussion

Among our study population in Dungarpur, Rajasthan, MGNREGA had a significant effect on reducing wasting and underweight among infants in households that participated in MGNREGA compared with households that did not. The only other study that assessed the effect of MGNREGA on child malnutrition showed a negative effect on stunting, but not on underweight and did not provide estimates for wasting [8]. Our study results are comparable to that of “The Chars Livelihood Programme” in Bangladesh, a cash-for-work programme, which was shown to be associated with reduction in all the three anthropometric indicators of malnutrition in children <5 years in the participating households [11]. However, unlike the Food Consumption Survey of Niger’s public works programme which showed that children 6 to 60 months were twice as likely to be malnourished in the participating households [32], there was reduced wasting and underweight among infants in the MGNREGA employed households even after controlling for factors that influences the propensity to participate into the programme.

The findings of the focus group discussions suggested that although MGNREGA may help avoid starvation, lower than standard wages and delayed payments meant that the participants did not receive all the benefits of the programme. Several studies and programme audit reports have identified such problems related to wages and timely payments, a majority of which were attributed to corruption [10]. Contrary to our hypotheses, results from path analysis did not show that household food security and infant feeding had an effect on infant malnutrition, but did suggest a pathway of effect through birth-weight. The probability of being born with low birth-weight (<2500 grams) was lower in participating households than in non-participating households, which reduced the risk of infant malnutrition in the participating households. This suggests the possibility that the benefits of the programme function through a pathway affecting women during pregnancy. However further investigation is needed to examine this.

In our study, the economic benefits of participating in MGNREGA appear to be short term which helps to prevent acute malnutrition, but insufficient to have an effect on chronic malnutrition. This argument is supported by our finding that the effect on the anthropometric indicators of malnutrition did not vary with the number of years of participation of the households in MGNREGA. Further, the findings of the focus group discussions suggested that although MGNREGA was able to prevent hunger, the earnings were inadequate to confer household food security. Even if food security was obtained, this is unlikely to translate into adequate infant feeding because of lack of appropriate knowledge about infant feeding among the mothers in the study population. Cultural practices, societal norms and poverty played an important role in influencing mothers’ knowledge and practices. Other studies in different parts of India have also highlighted the problem of inadequate knowledge among mothers and the misconceptions prevalent with regards to infant feeding and care [33], [34].

The path-models also provide an understanding of the role of the socioeconomic context in determining the effects of MGNREGA. MGNREGA is probably able to fulfil the basic nutritional requirements of pregnant women thereby reducing low birth-weight among infants in participating households compared with non-participating households in the poor socio-economic group. However, MGNREGA was not found to be effective in reducing malnutrition among infants in the comparatively better-off households. Although the results of path-analyses rely on the assumption of “no unmeasured confounding”, these are not causal models. Nevertheless, the results could have important implications for programme targeting.

### Limitations

Cross-sectional studies provide a snap-shot of a point in time and the anthropometric indicators fluctuate across infancy and childhood, however, the magnitude of the logistic regression results suggest that the observed findings provide reasonable evidence. Considering that MGNREGA-households were comparatively poor (a known risk factor of malnutrition), the logistic regression and path analysis results of protective effect of MGNREGA against malnutrition could not have been overestimated. Participation in MGNREGA is through self-selection and the exploratory regression analysis identified below poverty line status to be associated with household’s participation in MGNREGA in the study population. Although this factor was accounted for in the multivariable models, there could be other unknown factors that influenced participation in MGNREGA, thus selection bias cannot be completely ruled out. Further, despite adjusting for all known confounders identified from literature, there could still be residual confounding by unknown confounders.

While the path-analyses controlled for a few determinants of low birth-weight (such as maternal age, pregnancy desired, birth-order, caste, maternal education and gender) data on other important factors such as pre-pregnancy maternal nutrition and height, obstetric history such as age at first pregnancy, inter-pregnancy intervals etc. were not available. Since the study hypothesis was concerned with child related factors, data on the maternal factors were not collected. Including these factors in the model could alter the magnitude, strength and the direction of impact of MGNREGA on low birth-weight and thereby on infant malnutrition. Nevertheless, the study generates an important hypothesis about the positive effect of MGNREGA on infant nutrition through a maternal pathway which could be further explored in subsequent studies.

## Conclusion

This study is the first we know of to analyse the effect and the pathways of effect of MGNREGA on infant malnutrition and empirically demonstrates the inter-play of the various determinants of malnutrition. However, further studies are required to measure the effect of MGNREGA on infant and child malnutrition in different social, economic and geographical settings in India and also to delineate the observed maternal pathway. Ensuring timely and adequate payment could improve food security, and augment the protective effect of the MGNREGA. Factors such as lack of mothers’ knowledge about feeding and cultural practices related to inappropriate feeding are important risk factors of infant malnutrition. Identifying and addressing such factors, over and above the social and economic policies, is key in efforts to reduce malnutrition among infants. Therefore, policies need to focus on these factors and target the persistent problem of malnutrition prevalent in India through a convergence of development, health and nutrition policies and programmes.

## References

[1] International Institute for Population Sciences (IIPS) and Macro International (2007) National Family Health Survey (NFHS-3), 2005–06, India: Key Findings Mumbai: IIPS.

[2] Pelletier DL (1994) The relationship between child anthropometry and mortality in developing countries: Implications for policy, programs and future research. Journal of Nutrition 124: 2047S–81S. 7931716 10.1093/jn/124.suppl_10.2047S

[3] Vesel L, Bahl R, Martines J, Penny M, Bhandari N, et al (2010) Use of new World Health Organization child growth standards to assess how infant malnutrition relates to breastfeeding and mortality. Bull World Health Organ 88: 39–48. 20428352 10.2471/BLT.08.057901 PMC2802434

[4] Charmarbagwala R, Ranger M, Waddington H, White H (2004) The determinants of child health and nutrition: a meta-analysis: World Bank.

[5] Frongillo EA, de Onis M, Hanson KMP (1997) Socioeconomic and demographic factors are associated with worldwide patterns of stunting and wasting of children. The Journal of Nutrition 127: 2302–9. 9405578 10.1093/jn/127.12.2302

[6] Nair M, Webster P, Ariana P (2011) Impact of non-health policies on population health through the social determinants pathway. Bull World Health Organ 89: 778. 22084518 10.2471/BLT.11.093799 PMC3209731

[7] The National Rural Employment Guarantee Act, (5 September 2005, 2005).

[8] Uppal V (2009) Is the NREGS a Safety Net for Children? (Young Lives student paper). Oxford: Young Lives, University of Oxford.

[9] Dev SM (2011) NREGS and child being. Working paper WP-2011-004. Mumbai: Indira Gandhi Institute of Development Research.

[10] Khera R (2011) The Battle for Employment Guarantee: Oxford University Press.

[11] Mascie-Taylor CG, Marks MK, Goto R, Islam R (2010) Impact of a cash-for-work programme on food consumption and nutrition among women and children facing food insecurity in rural Bangladesh. Bull World Health Organ 88: 854–60. 21076567 10.2471/BLT.10.080994 PMC2971521

[12] Jha R, Bhattacharyya S, Gaiha R (2011) Social safety nets and nutrient deprivation: An analysis of the National Rural Employment Guarantee Program and the Public Distribution System in India. Journal of Asian Economics 22: 189–201.

[13] Burchi F (2010) Child nutrition in Mozambique in 2003: The role of mother’s schooling and nutrition knowledge. Economics & Human Biology 8: 331–45. 20646971 10.1016/j.ehb.2010.05.010

[14] Paul KH, Muti M, Khalfan SS, Humphrey JH, Caffarella R, et al (2011) Beyond food insecurity: how context can improve complementary feeding interventions. Food and nutrition bulletin 32: 244–53. 22073798 10.1177/156482651103200308

[15] Senarath U, Dibley MJ (2012) Complementary feeding practices in South Asia: analyses of recent national survey data by the South Asia Infant Feeding Research Network. Maternal & Child Nutrition 8: 5–10. 22168515 10.1111/j.1740-8709.2011.00371.x PMC6860754

[16] Government of Rajasthan and Planning Commission of India (2009) Dungarpur district human development report. Jaipur: Institute of Development Studies.

[17] Naandi Foundation (2011) HUNGaMA: Fighting hunger and malnutrition survey report. Hyderabad: Naandi Foundation.

[18] Gulliford MC, Adams G, Ukoumunne OC, Latinovic R, Chinn S, et al (2005) Intraclass correlation coefficient and outcome prevalence are associated in clustered binary data. Journal of clinical epidemiology 58: 246–51. 15718113 10.1016/j.jclinepi.2004.08.012

[19] World Health Organisation and Unicef (2009) WHO child growth standards and the identification of severe acute malnutrition in infants and children: a joint statement by the Word Health Organisation and the United Nations Children’s Fund. Geneva: World Health Organisation. 24809116

[20] Waterlow JC (1972) Classification and definition of protein-calorie malnutrition. British medical journal 3: 566–69. 4627051 10.1136/bmj.3.5826.566 PMC1785878

[21] De Onis M, Monteiro C, Akré J, Clugston G (1993) The worldwide magnitude of protein-energy malnutrition: an overview from the WHO Global Database on Child Growth. Bulletin-World Health Organisation 71: 703–14. PMC2393544 8313488

[22] de Onis M, Onyango AW, Van den Broeck J, Chumlea WC, Martorell R (2004) Measurement and standardization protocols for anthropometry used in the construction of a new international growth reference. Food Nutr Bull 25: S27–36. 15069917 10.1177/15648265040251S104

[23] World Health Organization (2006) WHO child growth standards : length/height-for-age, weight-for-age, weight-for-length, weight-for-height and body mass index-for-age : methods and development. Geneva, Switzerland.

[24] Swindale A, Bilinsky P (2006) Household Dietary Diversity Score (HDDS) for Measurement of Household Food Access: Indicator Guide (v.2). Washington, D.C: Food and Nutrition Technical Assistance Project, Academy for Educational Development.

[25] World Health Organisation (2010) Indicators for assessing infant and young child feeding practices. Part 2 Measurement. Geneva, Switzerland: WHO Press.

[26] Vasconcelos AGG, Varnier RM, Fonseca F (1998) The path analysis approach for the multivariate analysis of infant mortality data. Annals of epidemiology 8: 262–71. 9590605 10.1016/s1047-2797(97)00213-5

[27] Beauducel A, Herzberg PY (2006) On the performance of maximum likelihood versus means and variance adjusted weighted least squares estimation in CFA. Structural Equation Modeling 3: 186–203.

[28] Hooper D, Coughlan J, Mullen M (2008) Structural equation modelling: guidelines for determining model fit. The Electronic Journal of Business Research Methods 6: 53–60.

[29] Kenny DA, McCoach DB (2003) Effect of the number of variables on measures of fit in structural equation modeling. Structural Equation Modeling 10: 333–51.

[30] Planning Commission; Government of India (2012) Press Note on Poverty Estimates, 2009–10. Available: http://planningcommission.nic.in/news/press_pov1903.pdf. Accessed 2012 December 23.

[31] Asparouhov T, Muthén B (2010) Weighted least squares estimation with missing data. MplusTechnical Appendix.

[32] Webb P (1995) Employment programmes for food security in rural and urban Africa: experiences in Niger and Zimbabwe. In: Braun Jv, editor. Employment for poverty reduction and food security. Washington D. C.: International Food Policy Research Institute.

[33] Abbi R, Christian P, Gujaral S, Gopaldas T (1988) Mothers’ nutrition knowledge and child nutritional status in India. Food and nutrition bulletin 10: 51–4.

[34] Bawdekar M, Ladusingh L (2008) Contextual correlates of child malnutrition in rural Maharashtra. Journal of Biosocial Science 40: 771–86. 18241523 10.1017/S0021932008002757

[35] Baqui AH, Black RE, Yunus MD, Hoque ARA, Chowdhury HR, et al (1991) Methodological Issues in Diarrhoeal Diseases Epidemiology: Definition of Diarrhoeal Episodes. International journal of epidemiology 20: 1057–63. 1800404 10.1093/ije/20.4.1057

[36] World Health Organisation and Unicef (2006) Core questions on drinking-water and sanitation for household surveys. Geneva: WHO Press.

[37] International Institute for Population Sciences. District Level Household and Facility Survey - 3 (Reproductive and Child Health project). Mumbai: IIPS. Available: http://www.rchiips.org/ARCH-3.html. 2011 February 21.

[38] Chitnis S (1997) Definition of the terms scheduled castes and scheduled tribes: a crisis of ambivalence. In: VA Pai Panandiker, editor. The Politics of Backwardness: Reservation Policy in India. New Delhi, India: Centre for Policy Research.

[39] Galanter M (1984) Competing Equalities: Law and the Backward Classes in India. Berkeley, Calif: University of California Press.

[40] Demographic and Health Surveys. Available: http://www.measuredhs.com/aboutsurveys/dhs/questionnaires.cfm. 2011 February16.

[41] Vyas S, Kumaranayake L (2006) Constructing socio-economic status indices: how to use principal components analysis. Health Policy and Planning 21: 459–68. 17030551 10.1093/heapol/czl029

[42] Planning Commission; Government of India (2002) Tenth Five Year Plan: 2002–20007. New Delhi: Planning Commission of India.

